# Numerical Simulation of an Improved Updraft Biomass Gasifier Based on Aspen Plus

**DOI:** 10.3390/ijerph192417089

**Published:** 2022-12-19

**Authors:** Fugang Zhu, Laihong Shen, Pengcheng Xu, Haoran Yuan, Ming Hu, Jingwei Qi, Yong Chen

**Affiliations:** 1 Key Laboratory of Energy Thermal Conversion and Control of Ministry of Education, Southeast University, Nanjing 210096, China; 2 Everbright Greentech Technology Service (Jiangsu) Co., Ltd., Nanjing 211100, China; 3 Everbright Environmental Research Institute (Shenzhen) Co., Ltd., Shenzhen 518000, China; 4 Guangzhou Institute of Energy Conversion, Chinese Academy of Sciences, Guangzhou 510640, China

**Keywords:** biomass, improved updraft gasifier, simulation, Aspen Plus, gasification characteristics

## Abstract

In this paper, numerical investigation and optimization is conducted upon an improved updraft gasifier which is expected to overcome the weakness of conventional updraft gasifier. The comprehensive Aspen Plus model of the improved updraft gasifier is based on the RYield and RCSTR reactor. The tar prediction model is constructed, and the yield of tar is determined by the volatile of biomass and gasification temperature. The Aspen Plus simulation results agree very well with experiment results for the product yields and gasification efficiency, which shows the accuracy of the Aspen Plus model. The tar content in syngas of the improved gasifier is proved to be much lower than that of the conventional one by this model. The inflection point of the gasification efficiency occurs when air ratio is 0.25, and the optimum steam proportion in the air is 7.5%. Such a comprehensive investigation could provide necessary information for the optimal design and operation of the improved updraft gasifier.

## 1. Introduction

The depletion of fossil fuels and its impact on the global environment have become a major challenge facing the world [1,2]. Biomass is a widely used raw material that can provide energy and fuel at the same time [3,4,5,6]. Biomass has been thermochemically converted into different products, such as oil, char, and chemicals as well as syngas [7,8,9,10,11,12]. Among various thermochemical technology, gasification is considered as the most promising approach because the produced gas can be used in many fields [13,14,15,16,17].

There are many different kinds of gasification reactors, including fluidized-bed gasifier, updraft gasifier, and downdraft gasifier, etc. [18,19,20,21,22,23]. Compared with other gasifiers, updraft gasifier has higher gasification efficiency, wider adaptability of raw materials, and lower dust content in the syngas [24,25,26,27]. In updraft gasification, heat transfer and chemical reactions are known to be interrelated and occur simultaneously. Detailed experimental investigation of industrial gasifier has thus far been a challenging task due to the complex reactions taking place simultaneously and the lack of appropriate measuring and testing techniques. Therefore, numerical simulation becomes a useful tool to explore the complex processes in energy chemical industry.

Many Aspen Plus simulation [28,29,30,31,32,33] on updraft gasification have been carried out to investigate the effects of operating conditions on the gasification characteristics, such as temperature, biomass raw materials, types of gasification agents, and so on. Ismail et al. [34] constructed a two-dimensions simulation model using COMMENT code and investigated the gasification and combustion process in an updraft gasifier. The authors found that the predicted model was in good agreement with the experimental work. Cerinski et al. [35] proposed a pilot-scale biomass updraft gasifier model by combination of a pyrolysis kinetic model and a thermodynamic equilibrium model. In their study, pyrolysis process of biomass was modeled by kinetic mechanisms, and gasification process was modeled by minimization of Gibbs free energy approach. Umeki et al. [36] addressed the performance of updraft gasifier using a developed numerical model, which could predict experimental data successfully, and gasification reactions of char were further discussed. Yu et al. [37] implemented the reaction model (RXN model) based on comprehensive biomass gasification kinetics to predict the composition of syngas and tar, and the predicted data agreed with the experimental data well. Rosha et al. [38] employed Aspen Plus simulator to carry out the overall biomass pyrolysis system’s sensitivity analysis using a steady-state model and the effect of temperature for the product yield was discussed. The above research shows that the use of Aspen Plus simulation pushes the frontier of fundamental understanding of thermochemical interaction for the conventional updraft gasifier.

However, the updraft gasifier generally suffers from high tar content, which is likely to cause pipe blockage and gasifier shutdown. To lower the tar content in the product, an improved updraft gasifier is proposed by the researchers. In the novel updraft gasifier, the produced syngas is discharged from the reduction area, instead of the drying area in the conventional updraft gasification. As a result, the temperature of the syngas is higher, and the tar and dust content of the syngas is lower. For the novel updraft gasifier, there is no work related to the Aspen Plus simulation in the literature, which can enable reasonable theoretical prediction of various macroscopic phenomena.

This paper aims at numerical investigation and optimization of an improved updraft gasifier, which is expected to overcome the weakness of conventional updraft gasifier. For this purpose, a simulation model based on Aspen Plus is established, the model verification is performed based on the experiment investigation, and the effect of operating conditions on the product composition and gasification efficiency are predicted. Such a comprehensive investigation could provide necessary information for the optimal design and operation of the new gasification process.

## 2. Materials and Methods

### 2.1. Materials

The biomass studied in this work included corn stalk, wheat stalk, garden waste (which means grass and leaves collected from street cleaning), wood and fruit shell. The compositions of the five types of biomass were shown in Table 1, and the values of LHV are obtained from measurement. As seen in Table 1, the composition of various biomass is quite different. Corn straw, wheat straw and garden waste exhibit higher moisture and ash content, but lower volatile content, while wood and fruit shell exhibit lower moisture and ash content, but higher volatile content. The C/H/O contents of corn straw, wheat straw and garden waste are lower than that of wood and fruit shell.

### 2.2. Model Construction

**Figure 1:**
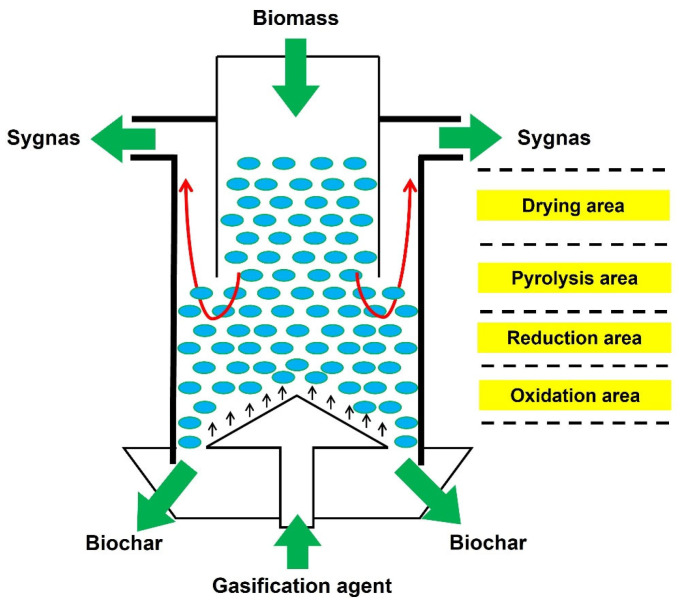
Schematic of improved updraft biomass gasifier.

The improved updraft biomass gasifier is shown in Figure 1. As we can see, biomass is fed from the top of the gasifier, and gasification agent is introduced from the bottom. From top to bottom, the gasifier can be divided into drying area, pyrolysis area, reduction area, and oxidation area. The syngas of the improved updraft gasifier is discharged from the reduction area rather than the drying area.

**Figure 2:**
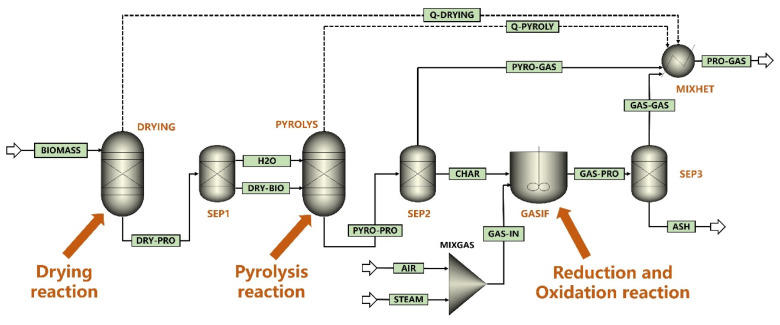
Schematic of Aspen Plus simulation process of improved updraft biomass gasifier.

The schematic of Aspen Plus simulation process of the improved updraft biomass gasifier is presented in Figure 2. It mainly includes three modules, in which drying reaction, pyrolysis reaction, reduction and oxidation reaction occur respectively. In the drying module, biomass is dried to generate water and dry biomass, which enter the pyrolysis module. In the pyrolysis module, dry biomass reacts to produce pyrolysis gas, tar, and coke. Pyrolysis gas, tar, and water are separated from the pyrolysis area of the gasifier. Coke and gasification agent react in the reduction and oxidation module. During the experiments, the biochar is collected from the ash outlet at the bottom of the gasifier, and the syngas and tar are discharged from the flue gas outlet at the top of the gasifier. The tar is recovered through ethanol washing firstly, and then the syngas is sent to the flue gas analyzer for composition analysis.

The main input parameters of the model are the proximate and ultimate analysis of the biomass, as shown in Table 1. The pyrolysis module is the RYield reactor block in Aspen Plus, which is used when the distribution of the product is known while the reaction kinetics is unknown. The product of the pyrolysis module includes CO, CO_2_, CH_4_, H_2_, H_2_O, O_2_, N_2_, C, S and ASH through user programed ‘PYROLYSIS’ FORTRAN statement with the proximate and ultimate analysis data. C_66_H_78_O_7.5_N is used as the model compound of tar, and the yield of tar is determined by the volatile of biomass and gasification temperature. The reduction and oxidation module is the RCSTR reactor block in Aspen Plus, which is used to simulate the gasification reaction of carbon and gasification agent. The reaction kinetic equations of carbon oxidation, hydrogen oxidation, steam gasification, steam reforming and so on are employed by user programed ‘GASIFI’ FORTRAN statement with reference data. The unreacted carbon and ash are discharged from the bottom of the gasifier.

### 2.3. Evaluation Indexes

In order to better analyze the gasification characteristics of biomass, several evaluation indexes are defined.

(1) Product yield

The product yield is defined as the quality of the product per kilogram of biomass gasification, as shown in Equation (1).

(1) $$Y_{p}=\frac{M_{p}}{m_{b}+m_{g}}\times 100\%$$

where $Y_{p}$ is the product yield, unit: %; $M_{p}$ is the quality of the product, unit: kg; $m_{b}$ is the quality of biomass, unit: kg; $m_{g}$ is the amount of air introduced by biomass gasification, unit: kg.

(2) Gasification efficiency

Gasification efficiency is defined as the ratio of the calorific value of the gas produced by biomass gasification to that of biomass, as shown in Equation (2).

(2) $$\delta =\frac{Q_{\mathrm{LHV},\;\mathrm{gas}}\times G_{g}}{Q_{\mathrm{LHV},\;\mathrm{bio}}\times m_{b}}\times 100\%$$

where δ is the gasification efficiency, unit: %; $Q_{\mathrm{LHV},\;\mathrm{gas}}$ is the calorific value of the gaseous product, unit: MJ/Nm^3^; $G_{g}\;$is the volume yield of the gaseous product, unit: Nm^3^/kg; $Q_{\mathrm{LHV},\;\mathrm{bio}}$is the calorific value of biomass, unit: MJ/kg.

(3) Air ratio

Air ratio is defined as the ratio of the amount of air introduced by biomass gasification to the theoretical amount of air required for complete combustion. The formula is shown in Equation (3).

(3) $$\mathrm{AR}=\frac{m_{g}}{m_{f}}$$

where *AR* is air ratio; $m_{f}$ is the theoretical amount of air required for complete combustion, unit: kg.

## 3. Results and Discussion

### 3.1. Model Validation

**Figure 3:**
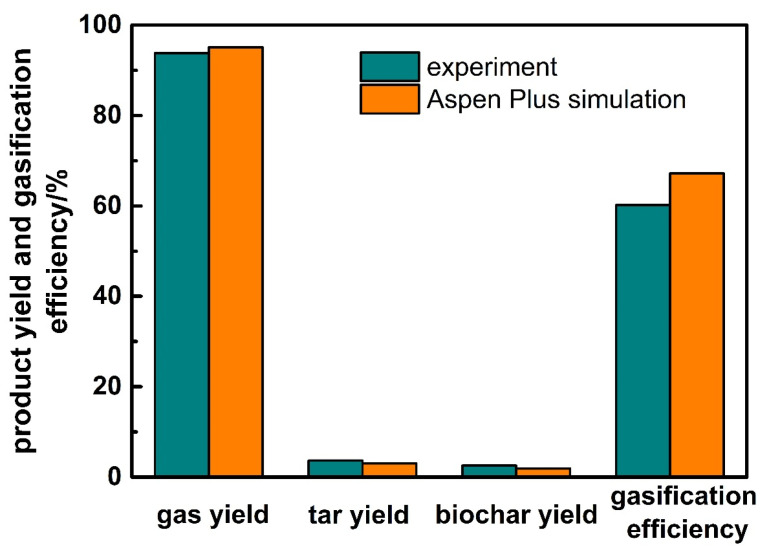
The comparison of product yield and gasification efficiency between experiment and Aspen Plus simulation results for the corn stalk.

**Figure 4:**
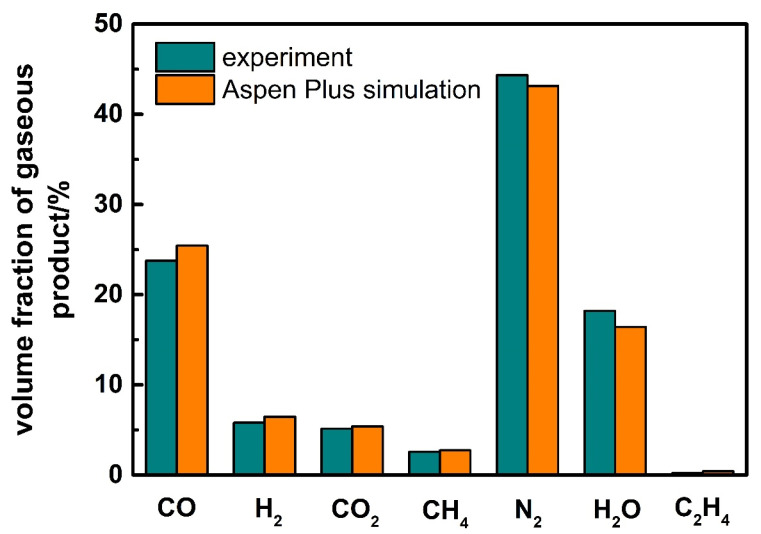
The comparison of gaseous product between experiment and Aspen Plus simulation results for the corn stalk.

Firstly, gasification experiments of corn stalk are carried out to verify the Aspen Plus simulation model. In experiments, feeding amount of corn stalk is 10 kg/h, air is used as the gasification agent with an *AR* of 0.28, and the gasification temperature is 800 °C. The comparison of product yield and gasification efficiency between experiment and Aspen Plus simulation results are shown in Figure 3. The yields of gas, tar, and biochar are 93.8%, 3.7%, and 2.5% respectively, and the gasification efficiency is 60.2%; the simulated product yield and gasification efficiency are 95.1%, 3.0%, 1.9%, and 67.2%, respectively. Figure 4 shows the comparison of gaseous product between experiment and simulation results. As we can see, the volume fraction of CO, H_2_, CO_2_, H_2_O, and N_2_ are 23.8%, 5.8%, 5.1%, 18.2%, and 44.3% in the experiment, while that of the simulation are 25.4%, 6.5%, 5.4%, 16.4%, and 43.1%, respectively.

Because some carbon is discharged from the gasifier without complete oxidation reaction due to insufficient reaction in the experiments, the gasification efficiency and the syngas yield of the simulation are more than that of experiments. The results of other types of biomass are similar to that of the corn stalk, so we do not show the results respectively here. In general, although there is a deviation between the simulation and experiment results, the deviation is less than 10%, which can be ignored. The simulation results are in good agreement with the experiment, which verifies the Aspen Plus model. We can use this model to simulate and optimize the improved updraft biomass gasifier later.

### 3.2. Comparison between Conventional and Improved Updraft Gasifier

**Figure 5:**
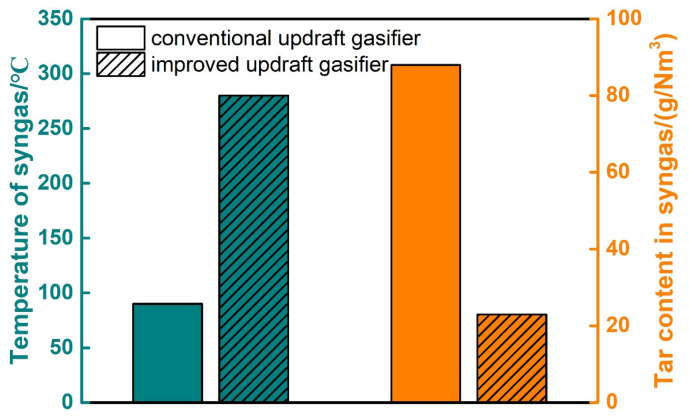
Comparison between conventional and improved updraft gasifier for the corn stalk.

We use this model to simulate the conventional and improved updraft gasifier. The syngas of the conventional updraft gasifier is discharged from the drying area, so the temperature of the syngas is set the same as the drying area. In our tar prediction model, the tar content is closely related to temperature. The comparison between these two types of gasifier are shown in Figure 5, and the results of different feedstock are similar, so only the results of corn stalk are shown here. The temperature of syngas of the conventional and improved updraft gasifier are 90 °C and 280 °C, and the tar content in syngas are 88 g/Nm^3^ and 23 g/Nm^3^ respectively. As the syngas of the improved updraft gasifier is discharged from the pyrolysis area rather than the drying area, its temperature is much higher than that of the conventional updraft gasifier. When the tar passes through the high-temperature pyrolysis area, the cracking reaction occurs to generate small molecular gas. As a result, the tar content in syngas of the improved gasifier is much lower than that of the conventional one. Through the improvement of the conventional updraft gasifier, the problem of high tar content in syngas can be solved, which shows the novelty and superiority of the conventional updraft gasifier.

### 3.3. Effect of Biomass Types

**Figure 6:**
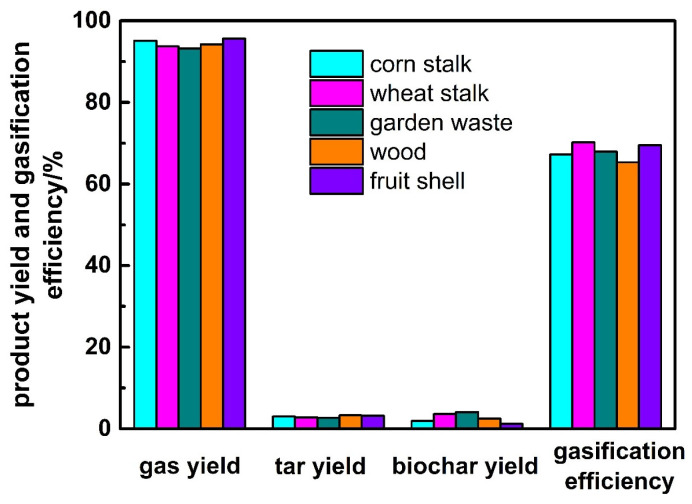
Effect of biomass types on product yield and gasification efficiency.

The effect of biomass types on product yield and gasification efficiency is shown in Figure 6. It is found that for various biomass, their gas yield is: fruit shell > corn stalk > wood > wheat stalk > garden waste, and biochar yield is the opposite. By comparing the composition of the five kinds of biomass in Table 1, we can find that the volatile content of biomass is: wood > fruit shell > corn stalk > wheat stalk > garden waste. The gas yield is basically proportional to the volatile content of biomass. However, it is not applicable for wood mainly because of its high carbon content, which leads to more carbon entering the ash. In addition, the gasification efficiency is: wheat stalk > fruit shell > garden waste > corn stalk > wood, with the highest of 70.2% and lowest of 65.3%.

**Figure 7:**
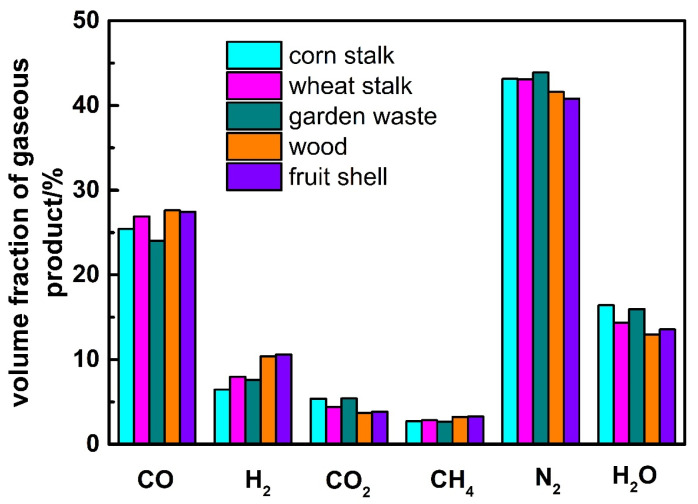
Effect of biomass types on gaseous product.

The gaseous products of the five kinds of biomass are shown in Figure 7. The volume fractions of CO and H_2_ in the gas product of wood and fruit shell are the highest whilst those of corn stalk and garden waste are the lowest. It can be concluded that the volume fractions of CO and H_2_ mainly depend on the carbon and hydrogen of biomass from Table 1. The carbon and hydrogen content of wood and fruit shell are much higher than that of corn stalk and garden waste.

### 3.4. Effect of Air Ratio

**Figure 8:**
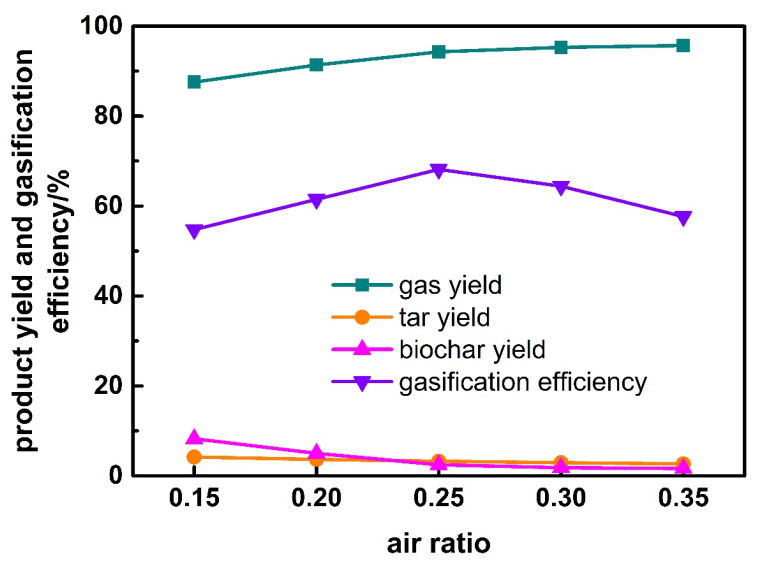
Effect of air ratio on product yield and gasification efficiency.

The effect of air ratio on product yield and gasification efficiency for corn stalk is presented in Figure 8. With the increase of air ratio, the gas yield increases while the tar and biochar yield decrease. Gasification efficiency increases firstly and then decreases, and the inflection point occurs at the air ratio of 0.25. It can be inferred that when air ratio is less than 0.25, there is still a large amount of carbon in corn stalk that does not react completely. The increase of air ratio leads to more carbon in corn stalk participates in the gasification reaction; therefore, the gas yield and gasification efficiency increase straightly. When air ratio continues to increase to more than 0.25, oxidation reaction between syngas and air occurs which results in the decrease of gasification efficiency and the increase of gas yield.

**Figure 9:**
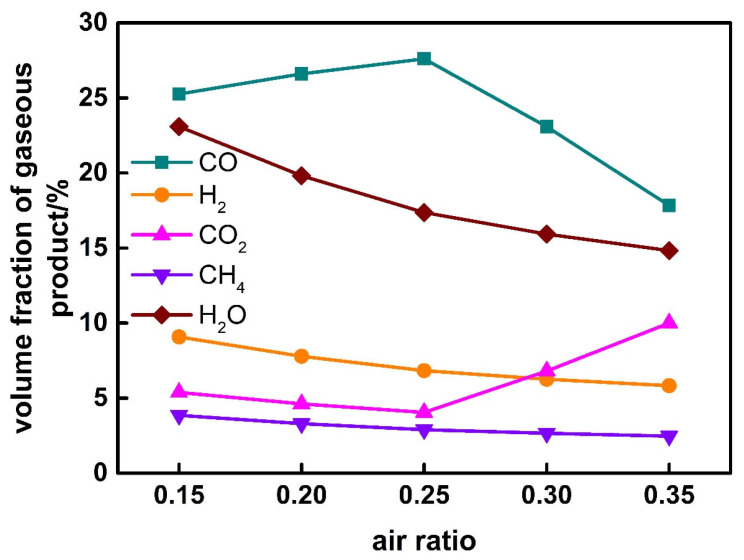
Effect of air ratio on gaseous product.

The effect of air ratio on gaseous product for corn stalk can be seen in Figure 9. With the increase of air ratio, the volume fraction of H_2_O, H_2_ and CH_4_ in gaseous product decreases. The volume fraction of CO increases firstly and then decreases; however, that of CO_2_ decreases firstly and then increases. The inflection point also occurs when the air ratio is about 0.25. The addition of air causes the combustion reactions of CO, H_2_ and CH_4_; thus, their volume fractions decrease. Due to the increase of carbon conversion, the volume fraction of CO increases firstly at the air ratio of less than 0.25.

### 3.5. Effect of Steam Proportion in Air

**Figure 10:**
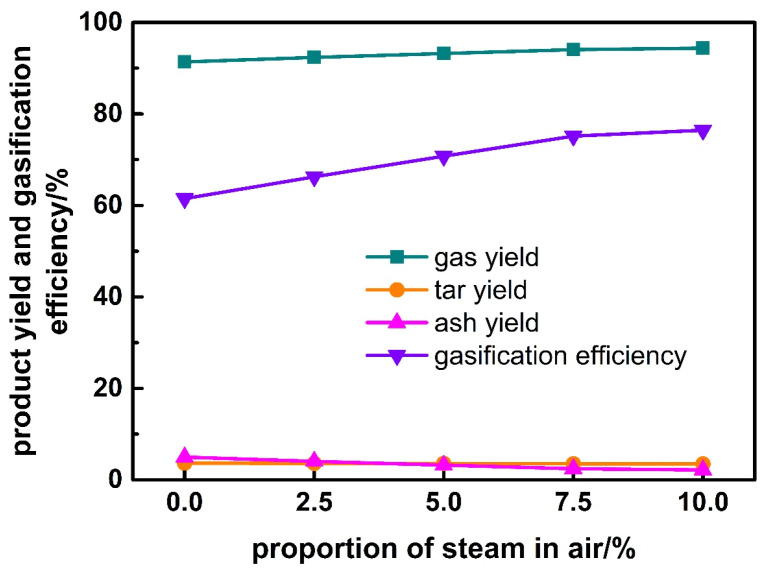
Effect of steam proportion in air on product yield and gasification efficiency.

**Figure 11:**
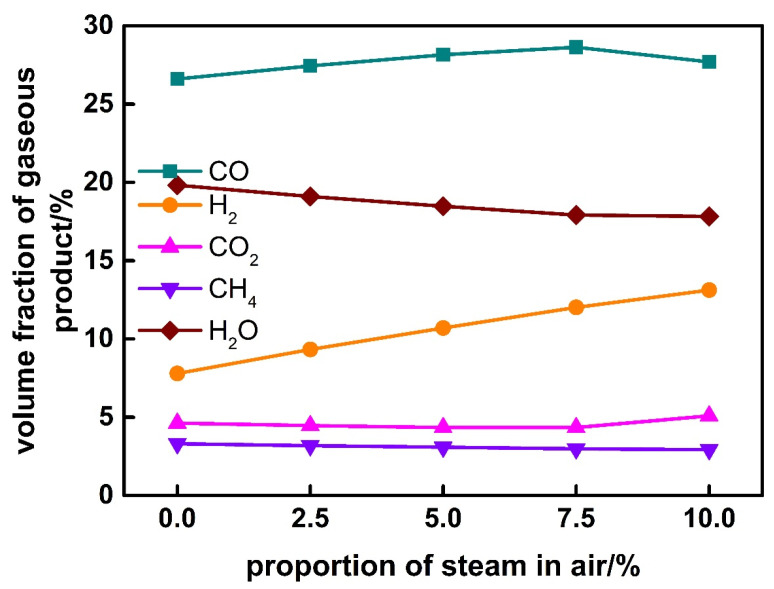
Effect of steam proportion in air on gaseous product.

The effect of steam proportion in air on the gasification characteristics of corn stalk is shown in Figure 10 and Figure 11. It is found that with the increase of steam proportion in air, gas yield increases while biochar yield decreases slightly; however, gasification efficiency increases firstly rapidly and then slowly. The volume fraction of H_2_ increases, that of CO increases firstly and then decreases, while that of CO_2_ decreases firstly and then increases. The inflection point occurs at the steam proportion of 7.5%.

The addition of steam in the air can promote the reaction between carbon and steam, which will reduce the content of unreacted carbon in ash, and increase the content of CO and H_2_ in gas products. Therefore, the gas yield, gasification efficiency, and the content of CO and H_2_ increase simultaneously. When steam proportion in the air increases to more than 7.5%, the carbon in biomass basically reacts completely. More steam will promote the reaction of CO and H_2_O to produce CO_2_ and H_2_, and for this reason the fraction of CO decreases and that of CO_2_ increases. The optimum steam proportion is 7.5%, which has higher gasification efficiency and more fraction of CO and H_2_ in the syngas.

## 4. Conclusions

The improved updraft gasifier is proposed with the intention of overcoming the weakness of the conventional updraft gasifier. For the novel updraft gasifier we proposed, there is no work related to the Aspen Plus simulation. In this work, a simulation model based on Aspen Plus is constructed to evaluate the performance of the improved updraft gasifier. Such a comprehensive investigation could provide necessary information for the optimal design and operation of the improved updraft gasifier. The following conclusions can be drawn from the previous study.

- A comprehensive Aspen Plus model of the improved updraft gasifier is constructed based on the RYield and RCSTR reactor. The tar prediction model is constructed, and the yield of tar is determined by the volatile of biomass and gasification temperature. The Aspen Plus simulation results agree very well with experiment results for the product yields and gasification efficiency, which shows the accuracy of the Aspen Plus model.
- The comparison between the conventional and improved updraft gasifier by this model shows that the tar content in syngas of the improved gasifier is much lower than that of the conventional one, which shows the novelty and superiority of the improved updraft gasifier.
- For the five different kinds of biomass, the gas yield is: fruit shell > corn stalk > wood > wheat stalk > garden waste, and biochar yield is the opposite. Wheat stalk has the highest efficiency of 70.2%, while wood has the lowest of 65.3%.
- With the increase of air ratio, the gas yield increases while the tar and biochar yield decrease. Gasification efficiency increases firstly and then decreases, and the inflection point occurs when air ratio is about 0.25, and the optimum steam proportion in the air is 7.5%.

## Figures and Tables

**Table 1:** The proximate and ultimate analysis of different types of biomass.

Components	Proximate Analysis (wt%)				Ultimate Analysis (wt%)					LHV (MJ/kg)
	M_ad_	A_ad_	V_ad_	FC_ad_	C_ad_	H_ad_	O_ad_	N_ad_	S_ad_	
Corn stalk	8.20	4.57	73.51	13.72	43.69	5.04	37.43	0.92	0.15	15.73
Wheat stalk	5.47	8.69	66.54	19.30	43.81	5.23	36.00	0.67	0.13	16.19
Garden waste	9.54	10.07	65.12	15.27	41.03	4.87	32.69	1.59	0.21	15.15
Wood	3.16	1.24	80.48	15.12	51.72	6.13	37.42	0.25	0.08	19.70
Fruit shell	3.63	0.82	76.95	18.60	49.43	6.34	39.42	0.29	0.07	18.91

## Data Availability

Not applicable.
